# Safety and Efficacy of Hepatitis B Vaccination in Cirrhosis of Liver

**DOI:** 10.1155/2013/196704

**Published:** 2013-06-06

**Authors:** D. Ajith Roni, Rama Mohan Pathapati, A. Sathish Kumar, Lalit Nihal, K. Sridhar, Sujith Tumkur Rajashekar

**Affiliations:** ^1^Medical Gastroenterology, Narayana Medical College Hospital, Nellore, Andhra Pradesh 524002, India; ^2^Clinical Pharmacology, Narayana Medical College Hospital, Nellore, Andhra Pradesh 524002, India

## Abstract

*Introduction*. Patients with chronic liver disease (CLD) are more likely to have severe morbidity and fatality rate due to superimposed acute or chronic hepatitis B (HBV) infection. The literature has shown that hepatitis B vaccines are safe and effective in patients with CLD, but the data in cirrhosis liver is lacking. We assessed the safety and immunogenicity of HBV vaccine in patients with cirrhosis liver. *Methods*. CTP classes A and B CLD patients negative for hepatitis B surface antigen and antibody to hepatitis B core antigen were included. All patients received three doses of hepatitis B vaccine 20 mcg intramuscularly at 0, 30, and 60 days. Anti-HBs antibody was measured after 120 days. *Results*. 52 patients with mean age 47.48 ± 9.37 years were studied. Response rates in CTP classes A and B were 88% and 33.3%. We observed that the alcoholic chronic liver disease had less antibody response (44%) than other causes of chronic liver disease such as cryptogenic 69% and HCV 75%. *Conclusions*. Patients with cirrhosis liver will have low antibody hepatitis B titers compared to general population. As the age and liver disease progress, the response rate for hepatitis B vaccination will still remain to be weaker.

## 1. Introduction

Globally, chronic HBV infection affects over 350 million people, and up to 40% of these cases may progress to cirrhosis, liver failure, or hepatocellular carcinoma [1]. Chronic liver disease [CLD] contributes to approximately 400000 hospitalizations and nearly 30,000 deaths annually worldwide [2, 3]. When compared with patients without liver disease, patients with CLD are more likely to have severe complications and also severe fatality rate due to superimposed acute or chronic HBV infection. Both acute and chronic coinfections with HBV can be prevented by HBV vaccination [4, 5]. Strong epidemiological evidence suggests an increased occurrence of fulminant liver failure, cirrhosis and hepatocellular carcinoma in patients with HBV, and HCV coinfection [6, 7]. HBV vaccination is safe and well tolerated and has high seroconversion rates in patients with mild to moderate CLD but has reduced efficacy in advanced liver disease and after liver transplantation [8–17]. To minimize the occurrence of HBV infection in CLD, a variety of organizations have recommended HBV vaccination for these patients [18, 19]. The immune response to HBV vaccines among patients with CLD varies from 70% to 90%. Hence in evaluating HBV vaccination in patients with cirrhosis liver, three questions need to be answered: (1) who needs vaccination? And (2) is the vaccination safe? and (3) is it effective? Answering these questions will provide an effective strategy for applying HBV vaccines in CLD. To this purpose we studied the safety and immunogenicity of 3 doses of 20 *μ*g of HBV vaccine in patients with cirrhosis liver.

## 2. Methods

This prospective open label study was conducted in the department of medical gastroenterology. Institutional ethics committee approved the study protocol. Informed consent was obtained from study participants. Patients with CLD of CTP class A and B were enrolled in the study. Both male and female patients between 18 and 60 years who were serologically negative for hepatitis B surface antigen, antibody to hepatitis B core antigen and have no history of hepatitis B vaccination were included. Patients with CTP class-C, having malignancy, acute liver disease, HIV, receiving immunosuppressive medications and life expectancy less than 120 days were excluded. All patients received three doses of HBV vaccine (Shanvac, M/s Shantha Biotech) 20 mcg intramuscularly over the deltoid region during all the three visits at 0, 30, and 60 days. A 5 mL of blood was collected in a plain vacutainer tube, the serum was separated, and the analysis for anti-Hbc total and titer for anti-Hbs was done on the same day of the collection of samples. Blood analysis for anti-Hbc total and titer for anti-HBs was done with chemiluminescence analyzer-Access 2 Immunoassay analyzer, and Beckman coulter. Anti-HBs antibody was measured after 120 days, and according to antibody titers patients were classified into good responders, poor responders, and nonresponders. Good responders were defined as those having the anti-HBs titer were > or =100 mUI/mL, poor responders having anti-HBs titer between 10 and 99 mUI/mL, and nonresponders having anti-HBs titer <10 mUI/mL. The secondary outcome was to assess the safety of HBV vaccination in CLD. Patients reported adverse events; infusion site reactions and routine laboratory parameters were considered safety markers of the study.

### 2.1. Statistical Analysis

Data was entered into excel spreadsheet 2007 and analyzed by using the GraphPad Prism Software version 4. All the continuous data will be expressed as mean ± SD. Categorical data was expressed as numbers and percentages. Chi-square test was used to detect differences between groups. A two-tailed *P* value < 0.05 was considered statistically significant. 

## 3. Results

**Figure 1:**
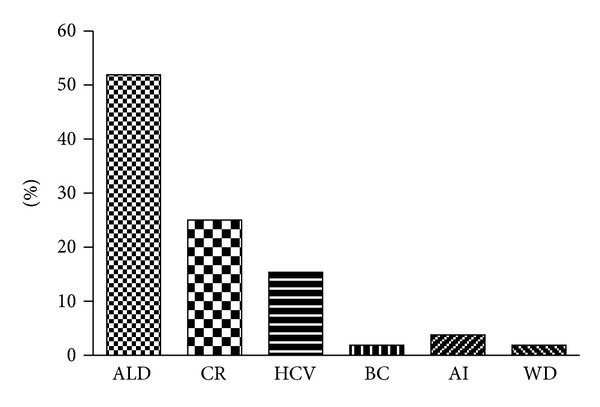
ALD: alcoholic liver disease; CR: cryptogenic hepatitis; HCV: hepatitis C virus; BC: budd chiarri; AI: autoimmune hepatitis; and WD: wilson's Disease.

**Figure 2:**
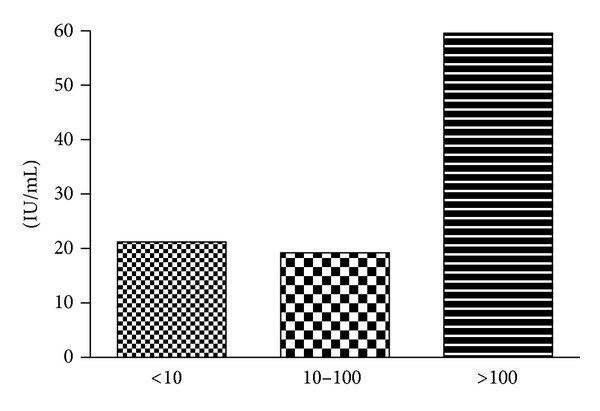
Antibody response shown in percentages after administration of HBV vaccine in chronic liver disease patients.

52 patients received 3 doses of HBV vaccine; the mean age of patients was 47.48 ± 9.37 years. There were 37 males and 15 females. 30 patients were less than 50 years of age and 22 were more than 50 years. 25 patients were in CTP class A and the rest 27 were in CTP class B. The reasons for CLD were alcohol 27/52 (52%), cryptogenic 13/52 (25%), hepatitis C 8/52 (15%), and others 4/52 (8%). (Figure 1). In this study 31/52 (60%) were good responders, 10/52 (19%) were of poor responders and 11/52 (21%) were nonresponders. (Figure 2). Patient characteristics and clinical profiles were shown in Table 1.

Among the patients who were less than 50 years of age, 21/30 (70%) of them were good responders, 6/30 (20%) were poor responders, and 3/30 (10%) were nonresponders. In patients more than 50 years, 9/22 (40%) were good responders, 5/22 (23%) were poor responders, and 8/22 (36%) were nonresponders. On comparing the responders rate between the age groups, patients in age group less than 50 years had significant responder rates (70%) than patients with age more than 50 years. (40%) (*P* = 0.03). 

We observed that 20/37 (54%) of the males and 11/15 (74%) of the females were good responders, 8/37 (22%) males and 2/15 (13%) females were poor responderss and nonresponders were 9/37 (24%) in males and 2/15 (13%) in females (*P* value 0.43). 

 When we were individually analyzing the vaccine response across different etiologies, we found that in 27 of the patients with alcohol induced CLD, 12/27 (44%) had good response, 7/27 (26%) had poor response, and 8/27 (30%) were non responders and in 8 patients with HCV related CLD 6/8 (76%) were good responders, 1/8 (12%) poor responders, and 1/8 (12%) were nonresponders. Overall response rates in 52 patients were as follows: 31/52 (60%) had good response, 10/52 (19%) had poor response, and 11/52 (21%) were nonresponders. (*P* = 0.36).

We also compared response rates with the child scores; out of 25 patients who were child A score, 22/25 (88%) were good responders, 1/25 (40%) were poor responders, and 2/25 (8%) were nonresponders and in child B class only 9/27 (33%) had good antibody response and 9/27 (33%) had poor response, 9/27 (33%) were nonresponders (*P* < 0.0001). None of our patients had suffered significant systemic or local adverse reactions. All patients complained of pain and redness during vaccine administration. No other adverse events were observed.

## 4. Discussion

 Vaccination with HBV vaccine is extremely safe in general population and in patients with chronic liver disease. The immunogenicity rates of vaccination in general population are >90% whereas in CLD it varied from 18% to 100%. In the present study, the response to standard HBV vaccination with a dose of 20 *μ*g at 0, 1, and 2 months in cirrhosis of liver with various etiologies was evaluated and compared. We found that good responders were only 60%, poor responders 19% percent, and nonresponders 21%. When we analyzed the various aetiologies of CLD and the vaccine response rate, we observed that the patients with alcoholic chronic liver disease (ALD) had poor antibody response (44%) as compared to other aetiologies of chronic liver disease such as cryptogenic (69%) and HCV (75%) related liver disease. Severity of the chronic liver disease predicts the response rate; the good responders were in CTP class A (88%) as compared to CTP class B (33.3%). It was observed that apart from severity of liver disease, the age of the patients also had contributed to antibody response; patients less than 50 years had a higher rate of response with hepatitis B vaccination than patients above 50 years. 

 Studies conducted by Lee et al., Wiedmann et al. and Keeffe and Krause [20–22] in CLD patients with HCV who had received 20 ug showed response rates of 100%, 89%, and 69%, respectively. However these studies did not include any patients with cirrhosis liver. In our study the good responders in patients with HCV related cirrhosis liver were 76%. However a fewer number of patients with HCV had participated in our study. In chronic ALD patients the response rates observed by Mendenhall et al., Bronowicki et al., and Rosman et al. [23–25] were 18%, 69%, and 46%, respectively. The previous studies had included only fewer cirrhotic patients. In our study the good responders in patients with alcoholic induced cirrhosis were only 44%, and all the patients were with child A or child B cirrhosis when compared to previous studies in the literature. 

 These observations suggest that if we are vaccinating at an earlier age (<50 years) and also at an early stage of chronic liver disease (child A), the immunogenicity of hepatitis B vaccination is superior as compared to patients with age more than 50 years and with child B cirrhosis liver. These observations suggest that if the patient is having advanced cirrhosis liver or in the age group of more than 50 years, it is always better to try different regimens like 40 ug or 80 ug of hepatitis vaccination or other routes of vaccine administrations like multiple intradermal dose. And the literature search had shown that the safety profile of these higher doses was comparable with that of normal dose. There were only few studies having data on multiple intradermal dose of hepatitis B vaccination in chronic liver disease, but it has not been yet approved in vaccination schedule.

## 5. Study Limitations

 We had evaluated only using standard dose of hepatitis B vaccine (Shanvac, M/s Shantha biotech) in patients with cirrhosis liver of CTP classes A and B only and had excluded CTP class C patients. However we have not compared the efficacy of Shanvac HBV vaccine with other available vaccines in the market. Our study populations were diversified and the subjects included in the different etiologic groups were small. We should have attempted with higher dose and/or weekly intradermal doses as per the evidence from the literature of patients with chronic kidney disease. Additionally long-term persistence of antibody titers, the frequency of estimating the postvaccination anti-Hbs titer, and the need for booster dose of hepatitis B vaccine were not evaluated due to technical and financial constraints. Another limitation of our study is that all the patients received the same quantity of HBV vaccine (20 *μ*g), and thus comparisons between high and low doses cannot be anticipated. 

## 6. Conclusions

 Patients with cirrhosis liver when compared to general population will have low postimmunization antibody titres against hepatitis B. As the age and the stage of liver disease progress, the immunogenicity of standard dose of hepatitis B vaccination against hepatitis B infection will still remain weak. Hence all the cirrhotics with non-HBV etiologies should be initiated on hepatitis B vaccination protocol at the time of diagnosis to achieve better protection against HBV.

 If the patient had not achieved seroconversion with standard universal dose of hepatitis B vaccine, reimmunization with a higher dose of hepatitis B vaccine schedule or with multiple intradermal route of vaccination may be considered. Further studies are needed to assess the antibody titres by considering type of vaccine, the dose to be administered, the route of administration, the frequency of antibody testing, and the requirement for booster dose.

## Figures and Tables

**Table 1:** Patient characteristics, clinical profiles, and immunological outcomes.

Antibody titers 226.88 ± 164 [IU/mL]	Total	Nonresponders [<10 IU/mL]	Partial responders [10–100 IU/mL]	Responders [>100 IU/mL]	*P* value
Age group 47.48 ± 9.37 [years]					
<50	30	3 [10%]	6 [20%]	21 [70%]	0.03
>50	22	8 [36%]	5 [23%]	9 [41%]	
Total	**52**	**11 [21%]**	**11 [21%]**	**30 [58%]**	
Gender					
Female	15	2 [13%]	2 [13%]	11 [74%]	0.43
Male	37	9 [24%]	8 [22%]	20 [54%]	
Total	**52**	**11 [21%]**	**10 [19%]**	**31 [60%]**	
Etiology					
Alcohol	27	8 [30%]	7 [26%]	12 [44%]	0.36
Hepatitis C	8	1 [12%]	1 [12%]	6 [76%]	
Cryptogenic	13	2 [15%]	2 [15%]	9 [70%]	
Others	4	0	0	4 [100%]	
Total	**52**	**11 [21%]**	**10 [19%]**	**31 [60%]**	
Child's class					
A	25	2 [8%]	1 [4%]	22 [88%]	<0.0001
B	27	9 [33.3%]	9 [33.3%]	9 [33.3%]	
Total	**52**	**11 [21%]**	**10 [19%]**	**31 [60%]**	
